# Human Milk from Tandem Feeding Dyads Does Not Differ in Metabolite and Metataxonomic Features When Compared to Single Nursling Dyads under Six Months of Age

**DOI:** 10.3390/metabo12111069

**Published:** 2022-11-04

**Authors:** Natalie S. Shenker, Alvaro Perdones-Montero, Adam Burke, Sarah Stickland, Julie A. K. McDonald, Simon J. S. Cameron

**Affiliations:** 1 Department of Surgery and Cancer, Imperial College London, London SW7 2AZ, UK; 2 Department of Metabolism, Digestion and Reproduction, Imperial College London, London SW7 2AZ, UK; 3 MRC Centre for Molecular Bacteriology and Infection, Imperial College London, London SW7 2AZ, UK; 4 Institute for Global Food Security, School of Biological Sciences, Queen’s University Belfast, Belfast BT9 5DL, UK

**Keywords:** human milk, lactation, metabolome, breastfeeding, infant feeding, microbiome, tandem feeding

## Abstract

Given the long-term advantages of exclusive breastfeeding to infants and their mothers, there is both an individual and public health benefit to its promotion and support. Data on the composition of human milk over the course of a full period of lactation for a single nursling is sparse, but data on human milk composition during tandem feeding (feeding children of different ages from different pregnancies) is almost entirely absent. This leaves an important knowledge gap that potentially endangers the ability of parents to make a fully informed choice on infant feeding. We compared the metataxonomic and metabolite fingerprints of human milk samples from 15 tandem feeding dyads to that collected from ten exclusively breastfeeding single nursling dyads where the nursling is under six months of age. Uniquely, our cohort also included three tandem feeding nursling dyads where each child showed a preferential side for feeding—allowing a direct comparison between human milk compositions for different aged nurslings. Across our analysis of volume, total fat, estimation of total microbial load, metabolite fingerprinting, and metataxonomics, we showed no statistically significant differences between tandem feeding and single nursling dyads. This included comparisons of preferential side nurslings of different ages. Together, our findings support the practice of tandem feeding of nurslings, even when feeding an infant under six months.

## 1. Introduction

According to ethnographic and anthropological research, a full term of lactation for a single nursling is estimated to range from over a year to up to seven years, with considerable variation between dyads. The average duration of breastfeeding for any one child is in the region of 4.2 years globally [1], but there is considerable geographical variability. For example, data from the last Infant Feeding Survey in the UK in 2010 showed only 55% of infants were given any breastmilk at six weeks postnatally, with 17% exclusively breastfed at three months; only 0.5% of mothers were breastfeeding at all at one year [2]. In the USA, 24% of infants in 2017 were still breastfed at six months [3]. In low and middle income countries, breastfeeding rates are improving, with a 2021 study of 57 countries indicating that 83% women were still breastfeeding at one year with 56.2% infants breastfed until at least 2 years [4]. Considering these ranges, the tandem breastfeeding of nurslings born from separate pregnancies is a natural occurrence [5]. Given the long-term advantages of exclusive breastfeeding to infants (cognitive development, reduced risk of child and adult obesity, and development of type 2 diabetes [6]) and their mothers (lower breast, ovarian, and endometrial cancer risk, decreased risk for development of hyperlipidaemia, and reduced risk of developing type 2 diabetes and other metabolic syndromes [7]), there is both an individual and public health benefit to its promotion and support. Therefore, a mother’s choice to undertake tandem feeding represents a natural outcome of such a promotion and support framework. Unless there is a contraindication to tandem feeding, and the new-born is meeting growth expectations, tandem feeding can be supported by healthcare professionals. However, some healthcare systems display a lack of clinical training and knowledge about the benefits of breastfeeding beyond six months exclusively [8], and there is a broader gap in evidence and knowledge of tandem feeding and the composition of human milk during such a period [9]. This leaves a void that risks parents not being able to make informed decisions about their infant feeding plans and ambitions.

We have previously shown that the metabolite and metataxonomic composition of human milk from dyads beyond six months of exclusive breastfeeding, compared to under this duration, does not significantly differ up to 24 months of lactation. There were however, some significant changes in individual metabolite features beyond this period up to the maximum of 48 months of lactation which may result from a concentration due to decreased feeding frequency [10]. This work complemented previous explorations of human milk composition beyond six months of exclusive breastfeeding that showed a concentration of several macronutrients including total protein, lactoferrin, and lysozymes [11]. However, the overall field of work in human milk composition beyond six months of exclusive breastfeeding has given conflicting results which may result from differences caused by other factors not included in analyses [12,13] and merits further attention. Research within the field of tandem feeding of nurslings born in different pregnancies represents an even smaller body of work which may be as a result of relative rarity of the practice or social stigma associated with the practice [5,14]. Macronutrients including total fat, protein, and carbohydrates during tandem feeding in a Polish cohort of 13 dyads showed no significant changes resulting from tandem feeding two children for more than a year [15]. However, as the importance of human milk components at a greater granularity of identity such as individual human milk oligosaccharides, fats, lipids, proteins, and microorganisms is realised, there is a need to move beyond high-level macronutrient analysis.

In this study, we aimed to begin to address this knowledge gap in human milk composition during tandem feeding by comparing the metabolite and metataxonomic fingerprints of tandem feeding dyads to human milk from exclusively breastfeeding dyads under six months of lactation. This cohort also, uniquely, included dyads where children from different pregnancies showed a preferential breast for feeding and thus allows a direct comparison between human milks. Recruitment was aided through participation of the Parenting Science Gang as previously reported [10]. As a result, we contribute what we believe to be the largest study of metabolite and metataxonomic composition of human milk from tandem feeding dyads to date.

## 2. Materials and Methods

This study was created with support from members of the Parenting Science Gang, who assisted with the recruitment of participants, preparation of study documents including the participant lifestyle questionnaire and informed consent forms, and the logistics of sample collection. This study utilises data from the three to six-month age group of our previously published work on duration of breastfeeding as a baseline comparator group [10]. Analysis of all samples was completed during the same experimental period.

### 2.1. Participant Recruitment

Members of the Parenting Science Gang were recruited through an online social media group. Participants were self-selecting based on tandem feeding. All participants provided written informed consent prior to sample donation and all information was link anonymised before analysis. No limitations were placed on participants with regard to lifestyle choices prior to study participation. Sample collection for this study was carried out as a sub-project (approval number R18006) of the Breastmilk Epigenetic Cohort Study under the Imperial College Healthcare Tissue Bank (HTA licence 12275), which received ethical approval from the Wales Regional Ethics Committee (reference 17/WA/0161). All procedures were carried out in accordance with the ethical standards of the Helsinki Declaration of the World Medical Association. Link anonymised lifestyle questionnaires were completed prior to sample donation. Lifestyle information was based on self-reported declarations of intake and not on a validated food frequency questionnaire.

### 2.2. Sample Collection, Handling, and Processing

All sample collection was completed on the same day at Charing Cross Hospital, Imperial College London, London, UK. Participants self-designated an hourly donation group between 09:00 and 16:00. Participants were asked to express the entire contents of either or both breasts using either hand expression, a manual pump which they supplied themselves, or an electric pump (Central Medical Supplies, Leek, UK). Prior to use, manual and electric pumps, tubing, and collection vessels were heat-sterilised using at-home, consumer-available microwave sterilisation bags. Participants were not restricted during the donation session and the breast was not sterilised prior to sampling. All participants donated a sample from both breasts and the sample with the largest volume was used for subsequent analysis in the single nursling dyad cohort but both were used for the tandem feeding cohort. Expressed samples were transferred to sterile 50 mL centrifuge tubes and transported to the analysis laboratory and processed within 30 min of donation. Samples were vortex mixed and separate aliquots were taken for metabolic fingerprinting analysis and DNA extraction. Aliquots for metabolic fingerprinting were analysed within one hour of donation and aliquots for DNA extraction were stored at −80 °C until required. The remaining volume was used for total volume and fat determination, and were stored at +4 °C until the day following collection for calculation of total volume and fat percentage. Sample volumes were determined through visual volumetric calculation using graduated 50 mL centrifuge tubes and total fat determined as a percentage of total volume after centrifugation at 3000× *g* for 5 min at +4 °C.

### 2.3. DNA Extraction and Metataxonomic Analysis

Using a sterile cotton swab, the fat layer from each 2 mL aliquot of human milk was removed after undergoing centrifugation for 10 min at 14,000× *g*. The sample pellet underwent DNA extraction using a FastDNA™ SPIN kit for soil (MP Biomedical, Irvine, CA, USA) following the manufacturer’s recommended protocol with the modification that bead beating was carried in three cycles at speed setting 6.0 with cooling on ice for 2 min between cycles. Extracted DNA was eluted into 50 µL of DNase-free water. The concentration of extracted dsDNA was calculated using the Qubit dsDNA broad range assay kit (ThermoFisher Scientific, Loughborough, UK) and a Qubit Fluorimeter 4.0 (ThermoFisher Scientific) with 1 µL of eluted DNA. Samples were extracted in batches consisting of 23 samples and one extraction negative using 2 mL of PCR grade water (Roche Diagnostics, Mannheim, Germany). Copy number for the 16S rRNA gene in each sample was determined using qPCR against gene copy number standards created through amplification of the full 16S rRNA gene of five randomly selected samples, as previously reported [16]. For metataxonomic analysis, DNA extracts were normalised to 5 ng/µL and sample libraries were created through amplification of the V1 to V2 region of the 16S rRNA gene as previously described [10]. The analysis pipeline is the same as previously described [10].

### 2.4. Metabolic Fingerprinting Using Laser Assisted Rapid Evaporative Ionisation Mass Spectrometry (LA-REIMS)

Within 1 h of sample donation, 2 mL of milk samples were placed into a 24 well tissue culture plate (Greiner Bio-One, Frickenhausen, Germany) and underwent centrifugation at 300× *g* for 10 min in a +4 °C chamber to produce a layer suitable for LA-REIMS analyses. Samples were analysed in donation batches so not all plate wells were filled in all runs. Each plate was placed on a raised platform within a Freedom Evo 75 (TECAN, Mannedorf, Switzerland) robot liquid handler adapted for high-throughput analysis as previously described [17]. Sample heating and evaporation utilised a helium-cooled 10.6 µM wavelength CO2 laser with fibre optic beam guide (FELS-25A, OmniGuide, Lexington, KY, USA) which was focused to a 500 µM spot size using a lens focusing system (Aesculight, Bothell, WA, USA), and analyte-containing vapor was taken into a Waters Xevo G2-XS quadrupole time-of-flight mass spectrometer fitted with a REIMS interface with t-piece sample infusion as previously described [10]. Due to the nature of LA-REIMS analysis, blank controls were not run as, for example, no solvents were used in extraction. Samples were analysed as a single replicate as due to the rapid nature of REIMS analysis, long-term batch effect of sample analysis is not typically observed [17]. For safety, LA-REIMS analysis was completed within a modified class two microbiological safety cabinet which had been adapted with suitable containment precautions matched to the 10.6 µM wavelength of the laser. All chemicals and solvents were handled in accordance with the material data safety sheet provided by their manufacturer.

### 2.5. Processing and Tentative Identifications of LA-REIMS Metabolic Fingerprinting Data

Raw data files for individual samples was imported into R studio (version 3.4.4) using a modified file reader (Waters Corporation) which also processed files to remove background signal and correct for mass drift according to the monoisotopic mass of leucine enkephalin ([M-H]^−^ mass 554.2615 and [M-H]^+^ mass 556.2712). From the processed spectra, individual masses were picked using the MALDIquant [18] package to a four decimal place accuracy as a consensus across all samples in which that feature was detected. A data matrix was created following total ion count normalisation and used in subsequent data analysis. Tentative metabolite identifications were obtained from a downloaded version of the Human Metabolome Database [19] using an in-house R script using a search threshold of a matched mass accuracy below 10 ppm. All features were classified against chemical taxonomy classifications. Tentative identifications are given in the supplementary material accompanying this manuscript for negative and positive ion detection mode data separately.

### 2.6. Statistical Analysis

Statistical analysis for metataxonomic and metabolic fingerprinting used the MicrobiomeAnalyst [20] and MetaboAnalyst [21] online pipelines, respectively, as previously described [10] with non-parametric tests used where assumptions for normality could not be met. Each data matrix was subjected to Log10 transformation and Pareto scaling after removal of low-variance variables based on standard deviation. A statistical cut-off of false discovery rate corrected *p* values in one-way analysis of variance univariate analysis was <0.05 and class differences were identified using Fisher’s LSD post hoc analysis. For analysis of participant phenotypic and demographic data in Table 1, data were imported into Prism 8 software (Version 8.1.2, GraphPad Software, San Diego, CA, USA). An Anderson–Darling normality test with a *p* value threshold of less than 0.05 was used to determine the appropriate univariate statistical test for quantitative variables and either a one-way ANOVA or Kruskal–Wallis with *p* value threshold of less than 0.05. Chi Square tests were used for categorical variables if they met the assumptions of the test—namely that all expected values are greater than 1 and at least 20% of the expected values are greater than 5. For expressed human milk volume, fat percentage, and estimation of bacterial load data, a non-normal distribution was observed for datasets using an Anderson–Darling normality test with the *p* value threshold of less than 0.05. As such, a Kruskal–Wallis test was used with a *p* value threshold of less than 0.05 with class differences identified using a post hoc Dunn’s multiple comparisons test with an adjusted *p* value threshold of less than 0.05. For preferential feeding side comparisons, a paired *t*-test was completed.

### 2.7. Availability of Raw Data

Raw 16S rRNA amplicon gene sequencing data are available through the European Nucleotide Archive under accession number PRJEB35510. Raw mass spectrometry data files are available through the MetaboLights repository under accession number MTBLS1370.

## 3. Results and Discussion

We utilised sample data from ten breastfeeding dyads where only a single nursling between three and six months of age from our previously published work [10]. This was complemented with samples from 15 tandem breastfeeding dyads with samples collected and analysed during the same period. Of the 15 tandem breastfeeding dyads, three reported that each different-aged nursling showed a preferential feeding side (left vs. right); allowing a direct comparison between human milk composition for different aged nurslings. As a result of the scale and mechanism of sample collection, it should be noted that we did not place restrictions on participants in terms of their feeding practices and did not control for time of day nor time since last feeding. As participants were asked to provide a full expression from each breast, we also did not separate between fore and hind milk in our analysis.

Participant demographic information is summarised in Table 1. Across the three participant groupings, statistically significant (*p* < 0.001) differences from Chi-squared tests were observed in ethnicity and diet, but not for age of mother, pre and post-pregnancy body mass index, nor gender of youngest nursling. It is important to note that dietary components, including fish consumption and vitamin C consumption, have previously been associated with individual components of human milk [22]. Due to the limited sample size in this study, we are restricted in our statistical power in exploring the effect of the reported differences in diet in Table 1. This should be considered a limitation of our study.

### 3.1. Fat, Volume, and Estimated Bacterial Load Does Not Differ between Human Milk from Tandem Feeding Dyads and Sole Nurslings

**Figure 1:**
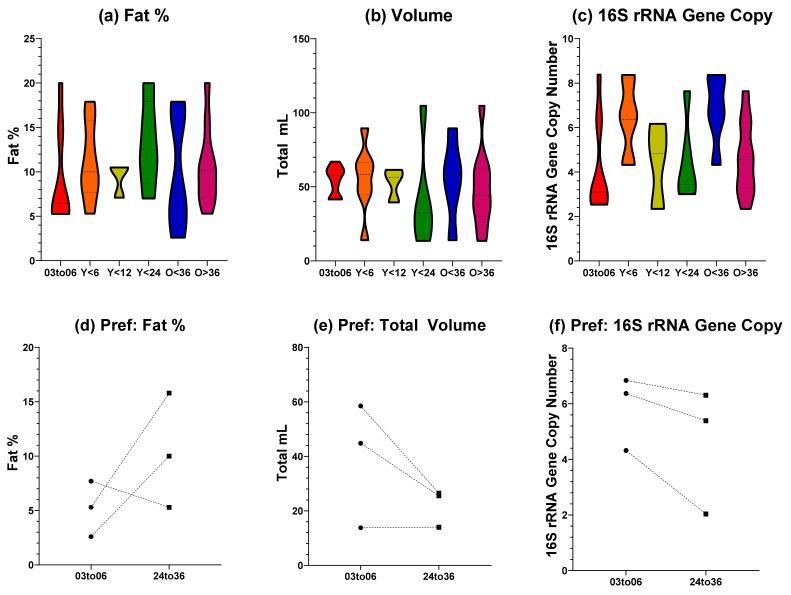
**Fat Percentage, Volume, and Estimated Bacterial Load of Human Milk**. (**a**) Total fat percentage of different groupings; (**b**) total volume of different groupings; (**c**) 16S rRNA gene copy qPCR for bacterial load for different groupings; (**d**–**f**) fat percentage, volume, and 16S rRNA gene copy for preferential side feeding. X axis labels indicate grouping: 03to06 = three to six months nursling age of exclusive breastfeeding dyads; Y < 6 = youngest tandem feeding nursling younger than six months; Y < 12 = youngest tandem feeding nursling younger than 12 months; Y < 24 = youngest tandem feeding nursling younger than 24 months; O < 36 = oldest tandem feeding nursling younger than 36 months; O > 36 = oldest tandem feeding nursling older than 36 months.

Total fat, volume, and 16S rRNA gene copy number values for the feeding side with the largest donation volume were compared across tandem feeding groups with single nursing dyads below six months of age, Figure 1a–c. Tandem feeding groups were categorised based on age of the youngest nursling (either below six, 12, or 24 months), or the age of the oldest nursling (either above or below 36 months). Total fat and 16S rRNA gene copy number values showed a normal distribution from a Shapiro–Wilk test (*p* > 0.05) and were analysed using a one-way ANOVA, whilst total volume showed a non-normal distribution (*p* < 0.05) and were analysed with a Kruskal–Wallis. For all three metrics, no significant differences (*p* > 0.05) were observed between all groups. As the study of human milk composition during tandem feeding is limited, there are few studies to compare our findings to. Macronutrients (such as fat, protein, and lactose) have previously been shown to be stable during tandem feeding until weaning, where there was a statistically significant change [15]. Other research, including our own [10], exploring natural term lactation beyond six months of exclusivity has parallels which are a useful comparison due to the typical overlap with nursing age. Macronutrient compositions of human milk beyond two years of lactation have shown stability up to 48 months, and show only small correlations with feeding frequency [13]. Similarly, levels of lactoferrin in human milk from breastfeeding dyads over 24 months of age show comparable levels to that seen from exclusively breastfeeding dyads under six months [23]. Taken together, this small body of research suggests that our findings fit the emerging narrative that macronutrients, that are also key providers of calorific intake, are stable in breastfeeding beyond the six months exclusive period and that this is consistent when tandem feeding is undertaken. It should be noted however, that our method of total volume and fat content was based on volumetric calculation which can have a large error rate attributed to it. This should, therefore, be considered a limitation of our study.

### 3.2. Metabolite Fingerprinting Using REIMS Shows no Multivariate or Univariate Differences for Dyad Groups

**Figure 2:**
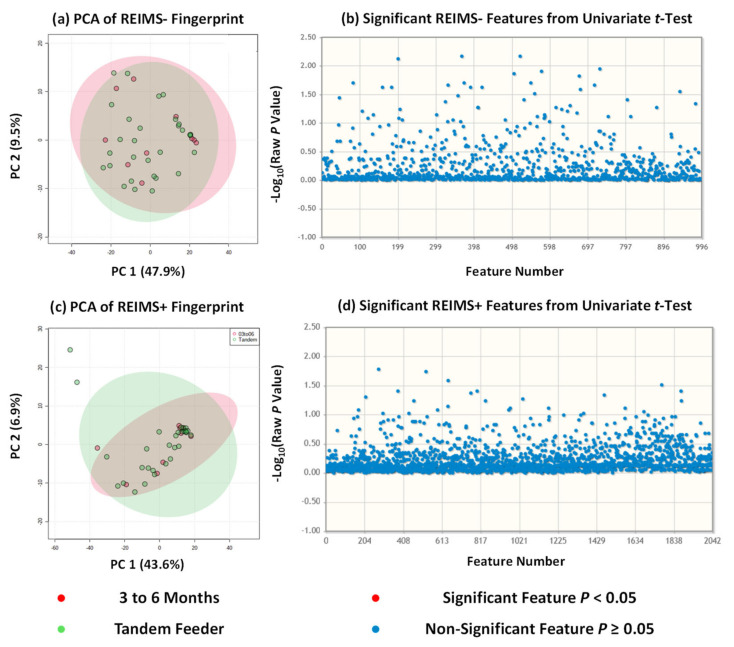
**REIMS Fingerprinting of Human Milk of Sole Tandem Feeding Nurslings**. (**a**) REIMS negative analysis of all tandem feeders against 3 to 6 month group; (**b**) REIMS negative univariate analysis showing no significant differences; (**c**) REIMS positive analysis of all tandem feeder against 3 to 6 month group; (**d**) REIMS positive univariate analysis showing no significant differences.

Rapid evaporative ionisation mass spectrometry (REIMS) is a technique within the ambient ionisation field that allows sample analysis without sample extraction and minimal preparative steps [24]. To date, it has been used to analyse a broad range of sample types from bacteria [25,26,27] and fungi [28,29] to clinical samples [30,31,32] and food items [17]. One of the main benefits of ambient ionisation methods is that, because of the lack of sample preparation, it allows analysis of a sample closer to its native form as compared to other methods for metabolite fingerprinting and/or metabolomics [33]. We have previously used REIMS in a partner study for the analysis of human milk composition from dyads beyond six months of exclusive breastfeeding [10]. Samples were analysed within 60 min of collection and thus removed the necessity for sample storage, freezing, and thawing which may alter the metabolic profile away from its initial state. We used the same approach in this study on milk composition from tandem feeding dyads with sample analysis repeated for both negative and positive ion detection modes. For statistical analysis, we utilised unsupervised multivariate analysis principal component analysis (PCA) to avoid the risk of overfitting due to limited sample size that accompanies supervised approaches, and univariate analysis using unpaired *t* tests. Across both approaches and ion detection modalities, no statistically significant separation or differences were observed, Figure 2, between human milk from breastfeeding dyads of a single nursling below six months and that from tandem feeding dyads. Our previous work suggested that there were a small number of metabolite features which were significantly altered beyond 24 months of lactation. In negative ion detection mode, these features were tentatively identified based on accurate mass as capryloyl choline, dipeptides, and 8-Hydroxy-6-docosanone; with the majority unable to be identified based on accurate mass against the HMDB collection. In positive ion detection mode, these features were more numerous and included complex lipids such as triacylglycerides and glycerophospholipids, epoxysiderol, triphenylenes, pol-g-D-glutamate, and Ferrioxamine B [10]. This may have some crossover with our tandem feeding cohort as it included a number of dyads where the oldest nursling is above 24 months of age. Albeit tentative, our work suggests that the metabolite composition of human milk reverts to that of milk for exclusively breastfed nurslings under six months of age.

**Figure 3:**
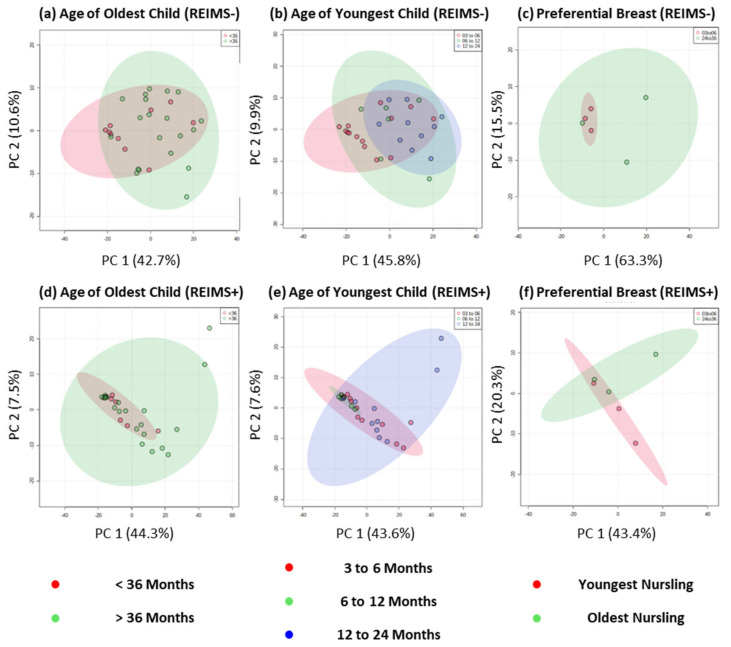
**REIMS Fingerprinting of Human Milk Based on Age and Preference of Tandem Feeding Nursling**. Metabolic fingerprinting of human milk based on negative ion detection mode data for (**a**) age of oldest child, (**b**) age of youngest child, and (**c**) milk from breast that is used solely to feed the youngest or oldest nursling. Metabolic fingerprinting of human milk based on positive ion detection mode data for (**d**) age of oldest child, (**e**) age of youngest child, and (**f**) milk from breast that is used solely to feed the youngest or oldest nursling.

To explore the milk composition of tandem feeding dyads further, we classified samples based on the age of the youngest and oldest child separately, Figure 3, and completed both multivariate PCA and univariate *t*-tests for two class comparisons and one-way ANOVA for more than two class comparisons. Across both types of analysis, and ion detection modes, no significantly different metabolite features were detected based on age of youngest or oldest child. This analysis approach allowed modelling of human milk composition based on age groupings which, due to sample size, would likely result in many false positive results if correlation analysis was completed [34]. Uniquely, this our sample cohort include three tandem feeding dyads that self-reported preferential side feeding for the youngest and oldest nursling. Previous work has shown that between-breast differences in human milk macronutrients (fat, lactose, protein, and overall energy contents) are not evident [35], but we believe our study is the first to analyse differences within tandem feeding infants and at the metabolite level. Across both ion detection modalities, no significant differences in multivariate or univariate analysis were detected. Albeit on a small number of samples, this analysis provides further support to the notion that the metabolite composition of human milk does not differ between that for exclusively breastfeeding a nursling under six months and tandem feeding nurslings regardless of age.

**Figure 4:**
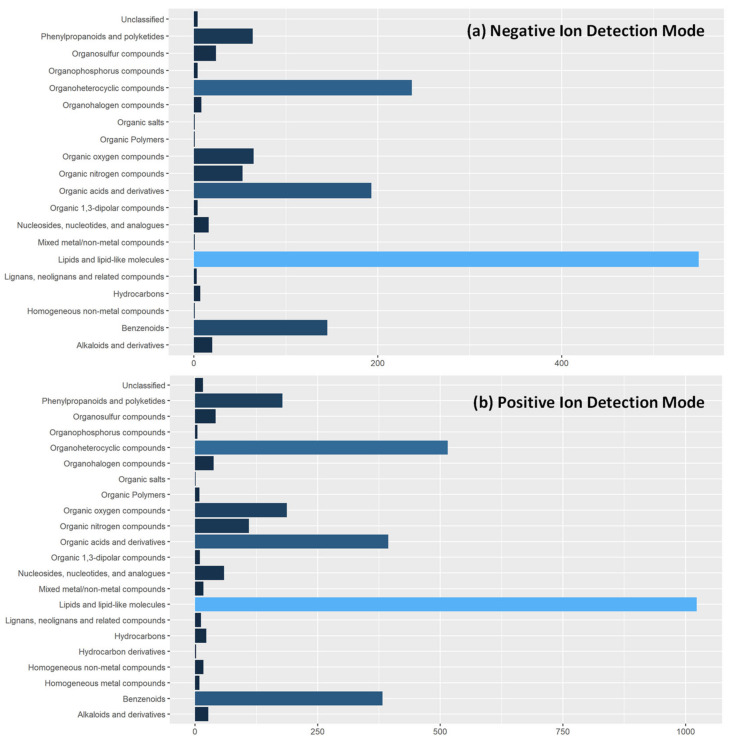
**Tentative Metabolite Identifications of Detected Features in REIMS Spectra**. Extracted features from REIMS mass spectra in both (**a**) negative ion detection mode and (**b**) positive ion detection mode were tentatively identified against the Human Metabolome Database using a cut-off of <10 ppm. Due to number of features in spectra, metabolites are classified according to the superclass level of chemical taxonomy in the HMDB collection to allow visual presentation.

In metabolite fingerprinting studies, it is typical that only significantly different features are identified to give insight into the biological and biochemical pathway underpinning [33]. As this is the first report of metabolite fingerprinting in tandem feeding dyads, we have added additional information on tentatively identified metabolite features in our data set. Through interrogation of the Human Metabolome Database with a cutoff threshold of <10 ppm, we identified 1400 and 3706 in negative and positive ion detection modes respectively. Due to the scale of features identified, we summarized their chemical taxonomy, Figure 4, at the superclass level. This gives a broad overview of metabolites detected using REIMS. Individual identifications are given in Supplementary Tables S1 and S2.

### 3.3. Metataxonomic Alpha or Beta Diversity Metrics Do Not Differ between Dyad Groups

The importance of the microbiome in early life is well established [36], although the mechanisms of its establishment are not [37]. Human milk composition appears to be important in supporting growth of keystone species [38], which are provided within the milk and are present even before the first feed [37]. To establish whether the human milk microbiome differs between tandem feeding and single nursling dyads, we used 16S rRNA gene amplicon sequencing. We have previously shown that this analytical run is free of ‘kitome’ [39] contamination [10] through the use of negative extraction controls. From our cohort, a total of 367,365 sequencing reads across 165 operational taxonomic units (OTUs) were obtained with an average of 9184 per sample (minimum 1032 to maximum 23,536). As there was a greater than ten-fold difference between minimum and maximum, libraries were rarefied to the smallest read number and then subjected to total sum scaling.

**Figure 5:**
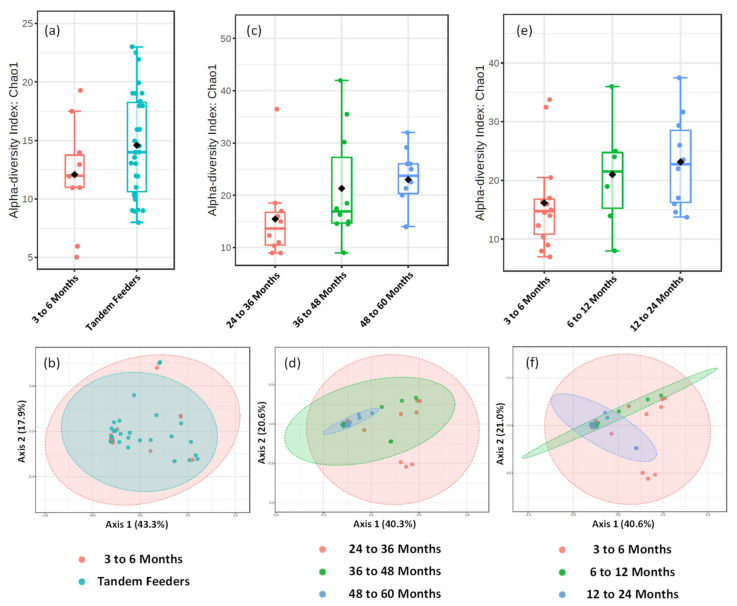
**Metataxonomic Profiling of Human Milk Based on Age of Tandem Feeding Nursling**. Metataxonomic analysis of human milk for 3 to 6 month nursling and tandem feeder nurslings using (**a**) Chao1 alpha diversity index (*p* value = 0.141) and (**b**) beta diversity (R^2^ = 0.021, *p* value = 0.556). Metataxonomic analysis of human milk based on age of oldest nursling using (**c**) Chao1 alpha diversity index (*p* value = 0.134) and (**d**) beta diversity (R^2^ = 0.197, *p* value = 0.005). Metataxonomic analysis of human milk based on age of youngest nursling using (**e**) Chao1 alpha diversity index (*p* = 0.137) and (**f**) beta diversity (R2 = 0.112, *p* Value = 0.085).

Direct comparison of alpha and beta-diversity metrics between tandem feeding and single nursling dyads, Figure 5a,b, show no significant differences (*p* > 0.05), and this is reflected at univariate analysis at the feature level of taxonomic classification. Further analysis of the tandem feeding cohort classified by age of the oldest nursling, Figure 5c,d, showed no significant (*p* > 0.05) differences in alpha-diversity measures, but a significant (*p* = 0.005) beta-diversity with an R_2_ value of 0.197. Within the 2D plot, tight clustering of the 48 to 60 month grouping can be observed, Figure 5d, which may suggest that there is less diversity as the age of the oldest child increases. However, there were no significant (*p* > 0.05) univariate differences at the feature level of OTU taxonomic classification, suggesting that these may be small-scale changes across a potentially broad range of taxons. To the best of our knowledge, the inclusion of the 48 to 60 month age group of nursling is the longest term of lactation that has been analysed with metataxonomic fingerprinting and thus, may be a dividing line for reduced inter-individual variation. It may also, however, be an artefact of a relatively small sample size. When samples are classified according to age of the youngest nursling, no significant (*p* > 0.05) differences in neither alpha nor beta-diversity were observed. However, a single OTU at the feature level assigned to the *Granulicatella* genus was significantly higher (*p* = 0.022) in the 6 to 12 and 12 to 24 month age group compared to the 3 to 6 month group. The genus *Granulicatella* has previously been found to be higher in human milk from overweight and obese mothers [40] but also in established breastfeeding from approximately 100 days postpartum [41]. It is typically considered a member of the oral microbiome but has been found in the lungs [42] and may therefore, be found in human milk in later lactation stages as a consequence of retrograde flux from the oral cavity [37].

## 4. Conclusions

Public health messaging around breastfeeding has largely focussed on the promotion and protection of exclusive breastfeeding for up to six months of life. However, the WHO’s recommendations is that breastfeeding continues for two years and beyond. Considering the duration of natural-term lactation, tandem feeding is a natural occurrence but one that is poorly studied. This has created an evidence gap in understanding whether the milk from tandem feeding an older nursling with an infant under six months maintains its composition. In this study, we have completed the most in-depth analysis of milk from the largest cohort of tandem feeding dyads and showed that there are no significant differences compared to the composition of human milk for single nursling dyads under six months. Although our study had limitations in terms of sample size and collection at a single time-point, it provides a foundation to support that nurslings under six months who are tandem fed with an older sibling born from a separate pregnancy are not disadvantaged due to an altered human milk composition.

## Figures and Tables

**Table 1:** **Participant demographic information**. Summary of participants present in each of three participant groups. Values are given either as counts or mean of group with corresponding standard deviation in brackets. For group count values, *p* value is given as output of Chi-Square test and for numerical values as the outcome of one-way ANOVA.

		Tandem Feeders	3 to 6 Months	Preferential Side	*p* Value
**Total Participants**		15	10	3	N/A
**Age (Years)**		25.3 (4.9)	26.9 (5.9)	27.4 (4.7)	0.675
**Ethnicity**	**Black-Caribbean**	1	0	0	<0.001
	**Latin American**	1	0	1	
	**White**	13	10	2	
**Body Mass Index**	**Pre-Pregnancy**	23.1 (3.6)	23.9 (5.5)	24.5 (5.2)	0.864
	**Post-Pregnancy**	25.3 (4.9)	23.9 (5.5)	24.5 (5.2)	0.786
**Gender**	**Boy**	11	4	3	0.131
	**Girl**	4	6	0	
**Diet**	**Meat Eater**	8	9	2	<0.001
	**Pescetarian**	1	1	0	
	**Vegetarian**	4	0	0	
	**Vegan**	2	0	1	

## Data Availability

Raw 16S rRNA amplicon gene sequencing data are available through the European Nucleotide Archive under accession number PRJEB35510. Raw mass spectrometry data files are available through the MetaboLights repository under accession number MTBLS1370.

## Supplementary Materials

The following supporting information can be downloaded at: https://www.mdpi.com/article/10.3390/metabo12111069/s1.

## References

[1] Bryant T. (2012). Tandem Nursing: A Review and Guidelines. Int. J. Childbirth Educ..

[2] Renfrew M., McAndrew F., Thompson J., Fellows L., Large A., Speed M. (2012). Infant Feeding Survey 2010.

[3] Gupta P.M., Perrine C.G., Chen J., Elam-Evans L.D., Flores-Ayala R. (2017). Monitoring the World Health Organization global target 2025 for exclusive breastfeeding: Experience from the United States. J. Hum. Lact..

[4] Wu H., Zhao M., Magnussen C.G., Xi B. (2021). Global prevalence of WHO infant feeding practices in 57 LMICs in 2010–2018 and time trends since 2000 for 44 LMICs. EClinicalMedicine.

[5] O’Rourke M.P., Spatz D.L. (2019). Women’s experiences with tandem breastfeeding. MCN Am. J. Matern. Child Nurs..

[6] Binns C., Lee M., Low W.Y. (2016). The long-term public health benefits of breastfeeding. Asia Pac. J. Public Health.

[7] Sattari M., Serwint J.R., Levine D.M. (2019). Maternal implications of breastfeeding: A review for the internist. Am. J. Med..

[8] Baranowska B., Malinowska M., Stanaszek E., Sys D., Bączek G., Doroszewska A., Tataj-Puzyna U., Rabijewski M. (2019). Extended breastfeeding in Poland: Knowledge of health care providers and attitudes on breastfeeding beyond infancy. J. Hum. Lact..

[9] Merše Lovrincevic K., Lepicnik V. (2018). tandem breastfeeding—Research on the knowledge of nursing and dietetics students. Obz. Zdr. Nege.

[10] Shenker N.S., Perdones-Montero A., Burke A., Stickland S., McDonald J.A., Alexander-Hardiman K., Flanagan J., Takats Z., Cameron S.J. (2020). Metabolomic and Metataxonomic Fingerprinting of Human Milk Suggests Compositional Stability over a Natural Term of Breastfeeding to 24 Months. Nutrients.

[11] Perrin M.T., Fogleman A.D., Newburg D.S., Allen J.C. (2017). A longitudinal study of human milk composition in the second year postpartum: Implications for human milk banking. Matern. Child Nutr..

[12] Verd S., Ginovart G., Calvo J., Ponce-Taylor J., Gaya A. (2018). Variation in the protein composition of human milk during extended lactation: A narrative review. Nutrients.

[13] Czosnykowska-Łukacka M., Królak-Olejnik B., Orczyk-Pawiłowicz M. (2018). Breast Milk Macronutrient Components in Prolonged Lactation. Nutrients.

[14] Jackson J.E., Hallam J. (2021). Against all odds—Why UK mothers’ breastfeeding beyond infancy are turning to their international peers for emotional and informative support. Health Care Women Int..

[15] Sinkiewicz-Darol E., Bernatowicz-Łojko U., Łubiech K., Adamczyk I., Twarużek M., Baranowska B., Skowron K., Spatz D.L. (2021). Tandem breastfeeding: A descriptive analysis of the nutritional value of milk when feeding a younger and older child. Nutrients.

[16] Cameron S., Huws S., Hegarty M.J., Smith D., Mur L.A.J. (2015). The human salivary microbiome exhibits temporal stability in bacterial diversity. FEMS Microbiol. Ecol..

[17] Cameron S.J., Perdones-Montero A., van Meulebroek L., Burke A., Alexander-Hardiman K., Simon D., Schaffer R., Balog J., Karancsi T., Rickards T. (2021). Sample Preparation Free Mass Spectrometry Using Laser-Assisted Rapid Evaporative Ionization Mass Spectrometry: Applications to Microbiology, Metabolic Biofluid Phenotyping, and Food Authenticity. J. Am. Soc. Mass Spectrom..

[18] Gibb S., Strimmer K. (2012). MALDIquant: A versatile R package for the analysis of mass spectrometry data. Bioinformatics.

[19] Wishart D.S., Jewison T., Guo A.C., Wilson M., Knox C., Liu Y., Djoumbou Y., Mandal R., Aziat F., Dong E. (2013). HMDB 3.0: The Human Metabolome Database in 2013. Nucleic Acids Res..

[20] Dhariwal A., Chong J., Habib S., King I.L., Agellon L.B., Xia J. (2017). MicrobiomeAnalyst: A web-based tool for comprehensive statistical, visual and meta-analysis of microbiome data. Nucleic Acids Res..

[21] Xia J., Sinelnikov I.V., Han B., Wishart D.S. (2015). MetaboAnalyst 3.0—Making Metabolomics More Meaningful. Nucleic Acids Res..

[22] Bravi F., Wiens F., Decarli A., Dal Pont A., Agostoni C., Ferraroni M. (2016). Impact of maternal nutrition on breast-milk composition: A systematic review. Am. J. Clin. Nutr..

[23] Czosnykowska-Łukacka M., Orczyk-Pawiłowicz M., Broers B., Królak-Olejnik B. (2019). Lactoferrin in Human Milk of Prolonged Lactation. Nutrients.

[24] Schäfer K.C., Dénes J., Albrecht K., Szaniszló T., Balog J., Skoumal R., Katona M., Tóth M., Balogh L., Takáts Z. (2009). In vivo, in situ tissue analysis using rapid evaporative ionization mass spectrometry. Angew. Chem. Int. Ed..

[25] Bardin E.E., Cameron S.J., Perdones-Montero A., Hardiman K., Bolt F., Alton E.W., Bush A., Davies J.C., Takáts Z. (2018). Metabolic Phenotyping and Strain Characterisation of *Pseudomonas aeruginosa* Isolates from Cystic Fibrosis Patients Using Rapid Evaporative Ionisation Mass Spectrometry. Sci. Rep..

[26] Cameron S.J., Bodai Z., Temelkuran B., Perdones-Montero A., Bolt F., Burke A., Alexander-Hardiman K., Salzet M., Fournier I., Rebec M. (2019). Utilisation of Ambient Laser Desorption Ionisation Mass Spectrometry (ALDI-MS) Improves Lipid-Based Microbial Species Level Identification. Sci. Rep..

[27] Bolt F., Cameron S.J., Karancsi T., Simon D., Schaffer R., Rickards T., Hardiman K., Burke A., Bodai Z., Perdones-Montero A. (2016). Automated High-Throughput Identification and Characterization of Clinically Important Bacteria and Fungi using Rapid Evaporative Ionization Mass Spectrometry. Anal. Chem..

[28] Gowers G.-O.F., Cameron S.J., Perdones-Montero A., Bell D., Chee S.M., Kern M., Tew D., Ellis T., Takáts Z. (2019). Off-Colony Screening of Biosynthetic Libraries by Rapid Laser-Enabled Mass Spectrometry. ACS Synth. Biol..

[29] Cameron S.J., Bolt F., Perdones-Montero A., Rickards T., Hardiman K., Abdolrasouli A., Burke A., Bodai Z., Karancsi T., Simon D. (2016). Rapid Evaporative Ionisation Mass Spectrometry (REIMS) Provides Accurate Direct from Culture Species Identification within the Genus Candida. Sci. Rep..

[30] Van Meulebroek L., Cameron S., Plekhova V., de Spiegeleer M., Wijnant K., Michels N., de Henauw S., Lapauw B., Takats Z., Vanhaecke L. (2020). Rapid LA-REIMS and comprehensive UHPLC-HRMS for metabolic phenotyping of feces. Talanta.

[31] Paraskevaidi M., Cameron S.J., Whelan E., Bowden S., Tzafetas M., Mitra A., Semertzidou A., Athanasiou A., Bennett P.R., MacIntyre D.A. (2020). Laser-assisted rapid evaporative ionisation mass spectrometry (LA-REIMS) as a metabolomics platform in cervical cancer screening. EBioMedicine.

[32] Cameron S.J., Alexander J.L., Bolt F., Burke A., Ashrafian H., Teare J., Marchesi J.R., Kinross J., Li J.V., Takats Z. (2019). Evaluation of Direct from Sample Metabolomics of Human Faeces using Rapid Evaporative Ionisation Mass Spectrometry (REIMS). Anal. Chem..

[33] Cameron S.J., Takáts Z. (2018). Mass Spectrometry Approaches to Metabolic Profiling of Microbial Communities within the Human Gastrointestinal Tract. Methods.

[34] Forstmeier W., Wagenmakers E.J., Parker T.H. (2017). Detecting and avoiding likely false-positive findings—A practical guide. Biol. Rev..

[35] Pines N., Mandel D., Mimouni F.B., Moran Lev H., Mangel L., Lubetzky R. (2016). The effect of between-breast differences on human milk macronutrients content. J. Perinatol..

[36] Pannaraj P.S., Li F., Cerini C., Bender J.M., Yang S., Rollie A., Adisetiyo H., Zabih S., Lincez P.J., Bittinger K. (2017). Association between breast milk bacterial communities and establishment and development of the infant gut microbiome. JAMA Pediatr..

[37] Moossavi S., Azad M.B. (2020). Origins of human milk microbiota: New evidence and arising questions. Gut Microb..

[38] Moossavi S., Sepehri S., Robertson B., Bode L., Goruk S., Field C.J., Lix L.M., de Souza R.J., Becker A.B., Mandhane P.J. (2019). Composition and variation of the human milk microbiota are influenced by maternal and early-life factors. Cell Host Microbe.

[39] Salter S.J., Cox M.J., Turek E.M., Calus S.T., Cookson W.O., Moffatt M.F., Turner P., Parkhill J., Loman N.J., Walker A.W. (2014). Reagent and Laboratory Contamination can Critically Impact Sequence-Based Microbiome Analyses. BMC Biol..

[40] Williams J.E., Carrothers J.M., Lackey K.A., Beatty N.F., York M.A., Brooker S.L., Shafii B., Price W.J., Settles M.L., McGuire M.A. (2017). Human milk microbial community structure is relatively stable and related to variations in macronutrient and micronutrient intakes in healthy lactating women. J. Nutr..

[41] Gonzalez E., Brereton N.J.B., Li C., Leyva L.L., Solomons N.W., Agellon L.B., Scott M.E., Koski K.G. (2021). Distinct Changes Occur in the Human Breast Milk Microbiome Between Early and Established Lactation in Breastfeeding Guatemalan Mothers. Front. Microbiol..

[42] Cameron S.J., Lewis K.E., Huws S.A., Hegarty M.J., Lewis P.D., Pachebat J.A., Mur L.A. (2017). A Pilot Study using Metagenomic Sequencing of the Sputum Microbiome Suggests Potential Bacterial Biomarkers for Lung Cancer. PLoS ONE.

